# Catheter directed diagnosis of ST-segment elevation myocardial infarction induced by type A aortic dissection: A case report

**DOI:** 10.1097/MD.0000000000018796

**Published:** 2020-01-17

**Authors:** Chi-Yao Huang, Yu-Po Hung, Tzu-Hsiang Lin, Szu-Ling Chang, Wen-Lieng Lee, Chih-Hung Lai

**Affiliations:** a Division of Interventional Cardiology, Cardiovascular Center, Taichung Veterans General Hospital, Taichung; b Department of Internal Medicine, Nantou Hospital, Nantou; c Institute of Clinical Medicine, National Yang-Ming University, Taipei; d Department of Anesthesiology, Taichung Veterans General Hospital, Taichung, Taiwan.

**Keywords:** aortic dissection, primary percutaneous coronary intervention, ST elevation myocardial infarction, type A aortic dissection

## Abstract

**Rationale:**

Type A aortic dissection (TAAD) is a life-threatening disorder yet it is hard to diagnose. The dissection might extend to the coronary artery causing ST-segment elevation myocardial infarction (STEMI). Physicians might not recognize this particularly early in its presentation and patients proceed to receive the primary percutaneous coronary intervention. We present such a case and found that the marked pressure difference between the radial and ascending aortae could be a useful clue for diagnosing the aortic dissection-related myocardial infarction.

**Patient concerns:**

A 58-year-old male was presented to our emergency department for the complaint of left side chest pain that lasted for an hour with concomitant hypotension. STEMI was diagnosed at that time.

**Diagnosis:**

The emergent primary percutaneous intervention was performed. When the diagnostic catheter was advanced to the ascending aorta, the systolic aorta pressure became 20 mm Hg higher than radial systolic pressure. Due to the abnormally large pressure differential between the peripheral radial artery and central ascending aorta, TAAD was suspected.

**Interventions:**

After angiography and computer tomography confirmed the diagnosis of TAAD, the patient was sent for emergent surgery.

**Outcomes:**

The patient was died because of extensive dissection and shock.

**Lessons:**

We present such a case and found that the marked pressure difference between the radial and ascending aortae during catheterization could be a useful clue for diagnosing the aortic dissection-related myocardial infarction. This clue had hinted our speedy examination of the occluded coronary artery and dissection flap, and led to an early and accurate diagnosis.

## Abstract 2

Supplemental Digital Content is available in the text

## 1 Introduction

Stanford type A aortic dissection (TAAD) is a life-threatening disease but is hard to diagnose. It has high mortality (18%–58%), and the mortality rate increases with time lapsed after symptom onset.^[1,2]^ In rare cases, the dissection site extends to the coronary artery (due either to hematoma compression or to torn coronary ostium or body) causing total coronary occlusion and ST-segment elevation myocardial infarction (STEMI). It is therefore important to diagnose as early as possible the STEMI that occurs secondary to TAAD. To shorten the “door-to-balloon” time, the required image examinations are often not done soon enough to diagnose the primary disorder. More frequently, TAAD is misdiagnosed as STEMI and treatment procedure taken is either the primary percutaneous coronary intervention (PCI) or thrombolytic therapy. TAAD dissection might occlude the ostium of coronary artery which slows down the procedure of PCI in searching the ostium of coronary artery. A prolonged procedure of PCI might hold making the correct diagnosis of TAAD and subsequently delaying the proper surgical therapy. Here, we present a case with the patient initially presented with STEMI and shock, and who received primary PCI thereafter. During the advancement of the diagnostic catheter, we found a marked pressure difference between the ascending aorta and the radial artery. This finding gave a hint for a possible TAAD. We then performed a manual aortogram which revealed the presence of an intimal flap, confirming the diagnosis of TAAD.

## 2 Consent for publication

Written informed consent was obtained from the patient's wife for publication of this case report and accompanying images.

## 3 Case presentation

**Figure 1:**
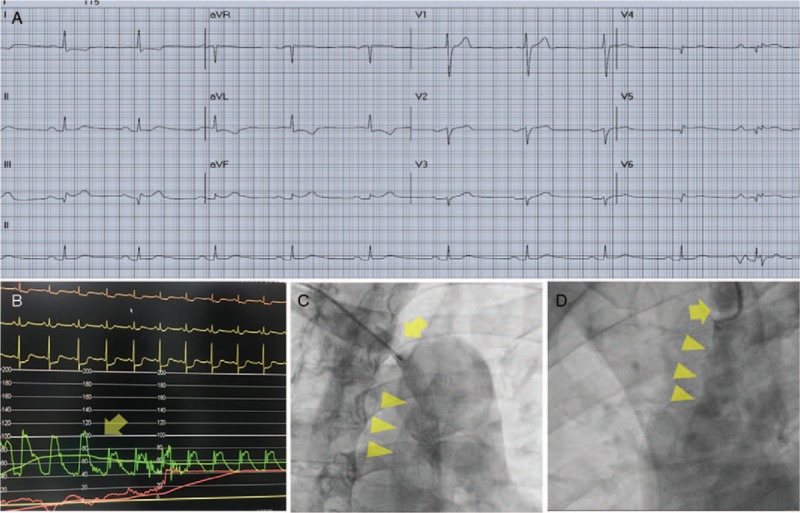
Electrocardiogram, hemodynamic change, and angiography of this TAAD related STEMI patient. (A) Right side 12-lead electrocardiogram showing inferior wall STEMI with right ventricle involvement (V4R with ST elevation). (B) While pulling the catheter from the ascending aorta to the innominate artery, pressure recordings show an apparent damping change (arrow). (C, D) Manual injection of contrast medium through the catheter showing an irregular lumen of the ascending aorta (arrowheads), suggesting a dissection. A stenosis is found near the tip of catheter (arrow), or the pressure damping site, suggesting a compromise of the true lumen by a false lumen. STEMI = ST-segment elevation myocardial infarction, TAAD = type A aortic dissection.

A 58-year-old male with hypertension without regular medical control was presented to our emergency department for the complaint of left side chest pain that lasted for an hour with concomitant hypotension. He had no cold sweating, radiating pain, or claudication. The 12-lead electrocardiogram suggested inferior wall and right ventricle STEMI, including ST elevation in leads III, and aVF, lead I with reciprocal ST depression, and right side V4R elevation (Fig. 1A). Under the impression of an inferior wall STEMI, antiplatelet medications and inotropic agent were administrated, and the patient was sent promptly for a primary PCI.

**Figure 2:**
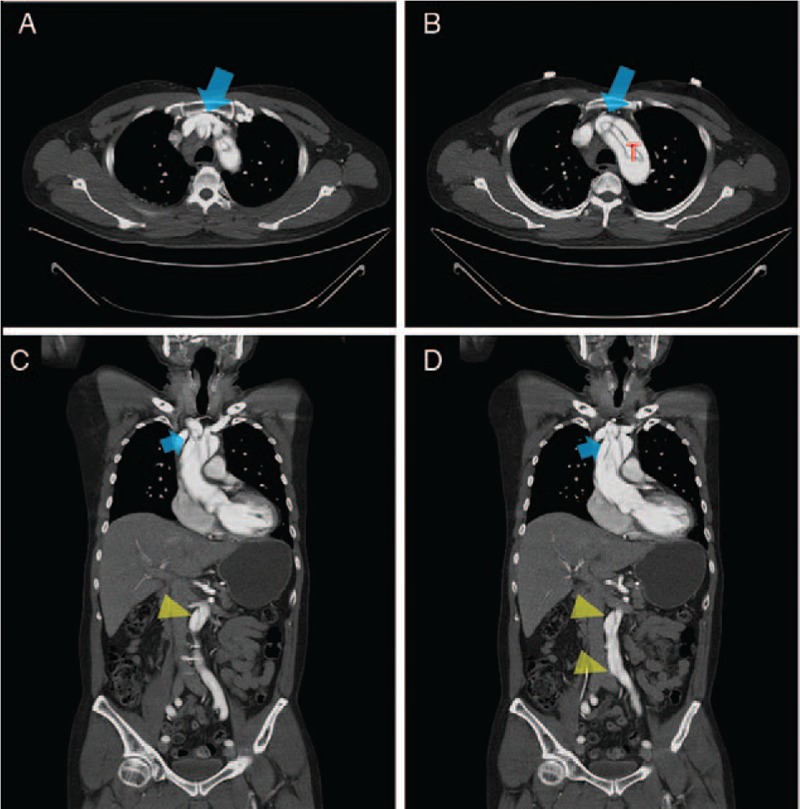
Serial images of computer tomography of aorta (AoCT). (A, B) AoCT showing TAAD and a small true lumen (T) compromised by a large false lumen in the ascending aorta, aorta arch, and proximal innominate artery (arrows). (C, D) AoCT showing severely compromised true lumen in the proximal innominate artery and at the junction between innominate artery and aortic arch (arrows). The dissection is seen extending to the abdominal aorta and iliac arteries (arrowheads). TAAD = type A aortic dissection.

Emergent primary PCI was approached through right radial access. After the insertion of radial sheath, the radial systolic blood pressure was 82 mm Hg. When the diagnostic catheter was advanced to the ascending aorta, the systolic aorta pressure became 20 mm Hg higher (ie, 102 mm Hg). Due to the abnormally large pressure differential between the peripheral radial artery and central ascending aorta, TAAD was suspected. After a short trial that failed to locate ostium of the right coronary artery, we withdrew the diagnostic catheter from the ascending aorta back to the innominate artery and there, we found the maximal pressure damping site around the junction of aorta arch and innominate artery (Fig. 1B). Through the diagnostic catheter, the injection of contrast medium was performed and subsequent imaging results showed a stenosis near the injection site and irregularly narrowing of the ascending aorta (Fig. 1C and D, Video 1). TAAD was finally diagnosed. The bedside echocardiography also conducted to visualize a dissection flap located over the ascending aorta together with a moderate aortic valve regurgitation. Computer tomography of the aorta further verified the TAAD with extensive dissection involving innominate artery, left carotid artery, and bilateral iliac arteries. Imaging results provided evidence of a false lumen compromising the innominate artery that had caused the pressure damping (Fig. 2A–D). The cardiovascular surgeon was consulted right away and the patient received aortic root and aortic valve replacement combined with bypass surgery thereafter. Due to the severe dissection and profounding shock, the patient passed away 2 days after the surgery.

## 4 Discussion

Only 2.5% of patients with TAAD has initial presentation as STEMI.^[2]^ In patients with initial diagnosis of STEMI and receiving primary PCI, 0.5% to 1.3% of these occurs secondary to TAAD.^[3,4]^ The incidence of both conditions (ie, STEMI and TAAD) is extremely rare. Since TAAD has a high mortality rate (estimated 1%–2% per hour increase immediately following symptom onsets in untreated patients), physicians should be aware of the possible diagnosis of TAAD in STEMI patients. Although careful physical examination and accurate history taking, such as tearing chest pain and radiating to back, pulse or/and blood pressure differentials, may help in the differential diagnosis of TAAD from true STEMI, it is still challenging as symptoms are mostly similar between these two. Moreover, the typical clinical presentation as well as radiographic findings might not always be present. On top of these, for STEMI patients, the required image studies (such as transthoracic echocardiography or computed tomography) may not be indicated initially, because the imaging procedure may cause delay and prolong the “door-to-balloon” time. Therefore, some STEMI patients with TAAD might proceed to receive primary PCI, which might not readily detect TAAD. It is, therefore, crucial to increase the awareness of cardiologists during primary PCI regarding clues of TAAD related STEMI.

In a study by Wang et al, increased resistance in PCI during physical advancement of the diagnostic catheter was felt in 60% of cases with STEMI secondary to TAAD.^[4]^ If the catheter were placed in the false lumen, greater resistance will be experienced. However, if the catheter were instead placed in the true lumen, the resistance will appear low or even be free of resistance. Nowadays, the radial rather than the femoral approach is used for primary PCI and the chance of driving the catheter into the true lumen is higher. For this reason, the diagnosis of TAAD is even more difficult to make. In our case, there is no resistance while advancing the catheter. It was only the marked pressure difference between the radial artery and the ascending aorta that had hinted us the possibility of TAAD. We withdrew the catheter to locate the site with the greatest pressure drop and found it near the junction between the aorta arch and innominate artery. The aortography, conducted with manual injection of contrast medium, showed a dissection flap over the ascending aorta, thus confirming the diagnosis of TAAD. The false lumen of TAAD could compromise the true lumen of major branches (such as innominate artery) making the pressure drop. Therefore, the pressure differential we found could be a useful and time-saving clue of TTAD related STEMI. In addition to the pressure changes observed during PCI, other phenomena also occur and are helpful. For example, following the injection of contrast medium into the aorta, the following might be observed:

(1) a discrepancy in diameters between the aortic root and the ascending aorta (100% occurrence),
(2) presence of intimal flap in the ascending aorta (75%), and
(3) the disappearance of the coronary artery ostium (40%).

All of the above findings are important diagnostic auxiliary clues for TAAD related STEMI.^[4]^ While these clues may not present themselves simultaneously in any individual patient, physicians should be cautious of these signs to save time in searching the occluded ostium of coronary artery and to lower the risk of further damages to the dissected vessel. The sooner the diagnosis of TAAD is made, the better is the survival chance.

Catheter directed a large blood pressure difference between the radial and ascending aorta is a helpful clue for the diagnosis of TAAD related STEMI.

## Author contributions

**Data curation:** Yu-Po Hung.

**Formal analysis:** Tzu-Hsiang Lin, Szu-Ling Chang.

**Writing – original draft:** Chi-Yao Huang.

**Writing – review and editing:** Wen-Lieng Lee, Chih Hung Lai.

Chih Hung Lai orcid: 0000-0003-1409-4979.

## Supplementary Material

Supplemental Digital Content

## References

[1] Tsai TT Nienaber CA Eagle KA Acute aortic syndromes. Circulation 2005;112:3802–13. 16344407 10.1161/CIRCULATIONAHA.105.534198

[2] Luo JL Wu CK Lin YH Type A aortic dissection manifesting as acute myocardial infarction: still a lesson to learn. Acta Cardiol 2009;64:499–504. 19725443 10.2143/AC.64.4.2041615

[3] Zhu QY Tai S Tang L STEMI could be the primary presentation of acute aortic dissection. Am J Emerg Med 2017;35:1713–7. 28511806 10.1016/j.ajem.2017.05.010

[4] Wang JL Chen CC Wang CY Acute type A aortic dissection presenting as ST-segment elevation myocardial infarction referred for primary percutaneous coronary intervention. Acta Cardiol Sin 2016;32:265–72. 27274166 10.6515/ACS20150424J PMC4884753

